# Phenomics and transcriptomic profiling of fruit development in distinct apple varieties

**DOI:** 10.1038/s41597-024-03220-4

**Published:** 2024-04-16

**Authors:** Weihan Zhang, Yuepeng Han, Liao Liao

**Affiliations:** 1 State Key Laboratory of Plant Diversity and Specialty Crops, Wuhan Botanical Garden, Chinese Academy of Sciences, Wuhan, 430074 China; 2 Sino-African Joint Research Center, Chinese Academy of Sciences, Wuhan, 430074 China

**Keywords:** Genomics, Plant sciences

## Abstract

Apple is one of the most economically important and popular temperate fruit trees. The domestication of apple has resulted in substantial phenotypic differences, particularly between wild and cultivated varieties. However, the relationship between gene expression and phenotypic variations in apple remains poorly understood. Here, we present a comprehensive dataset featuring five distinct apple varieties, including two wild varieties and three representative cultivated varieties. The dataset comprises of both phenomics data, encompassing twelve fruit quality-related traits continuously measured over two years, and transcriptomic data obtained at different developmental stages with three biological replicates. We performed basic quality control process, gene expression normalization and differential gene expression analysis to demonstrate the utility and reliability of the dataset. Our findings indicate that gene expression strongly related with phenotypic variations in apple. This dataset serves as a valuable resource, encompassing phenomics and transcriptomic data in multiple formats, thereby facilitating further exploration of the relationships between gene expression and phenotypic traits in apple.

## Background & Summary

Apple, scientifically known as *Malus* × *domestica* Borkh., is one of the most extensively cultivated and economically significant fruit crops worldwide. It belongs to the genus Malus within the *Rosaceae* family. Apples are temperate fruit trees that have been under cultivation for thousands of years^1^. With diverse range of cultivars and wild varieties, apple exhibit remarkable genetic and phenotypic variation^2–4^. This inherent diversity makes apple an intriguing subject for omics research. Moreover, the agricultural, economic, and nutritional importance of apples further emphasizes the need to unravel the underlying genetic mechanisms that govern various traits such as fruit quality, disease resistance, and yield. Understanding the underlying genetic mechanisms that drive phenotypic variations is crucial in apple genomics research.

Gene expression, which is the process of converting genetic information is converted into functional molecules such as proteins, plays a central role in regulating key traits in apples and other organism^5–8^. However, studying gene expression alone is insufficient. Phenotype data, encompassing the observable traits and characteristics of an organism, are crucial for establishing the link between gene expression and the phenotype. The association between gene expression and phenotypic variation constitutes a critical area of genetics study and molecular breeding, as it provides insight into the molecular basis of desirable traits^9^. By integrating gene expression data with phenotypic information, researchers can gain a deeper understanding of the underlying mechanisms that contribute to an organism’s observable features^2,3^. Moreover, selecting appropriate research materials, such as samples or developmental stages, is vital in obtaining reliable and meaningful data for accurate interpretation and meaningful insights into the relationship between gene expression and phenotype^10^.

Although some studies have been conducted on gene expression and phenotype variations in apples^6–8,11–16^. there are several limitations in terms of data quality and completeness. One key limitation is the absence of readily apparent phenotypic differences between samples, which impedes the identification of significant gene expression variations. Furthermore, the lack of continuous and comprehensive phenotypic data poses a hindrance to conducting thorough analyses and comparisons. Additionally, the inadequate coverage of sample developmental stages increases the likelihood of overlooking crucial gene expression changes at specific stages. Consequently, it is imperative to collect a comprehensive apple dataset that overcomes the above problems to investigate the relationship between apple gene expression and phenotypic variations.

**Fig. 1:**
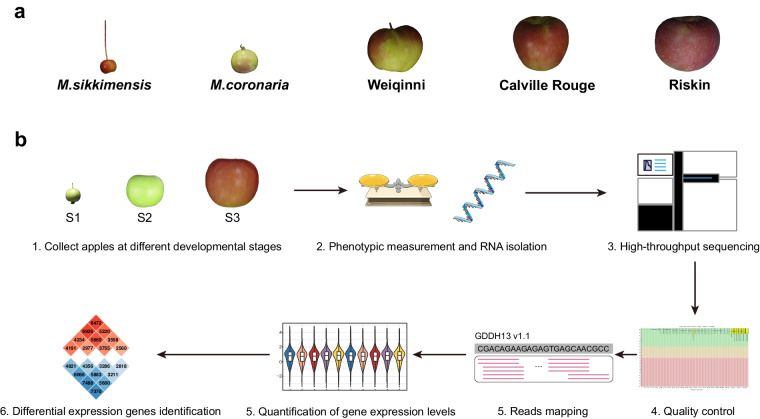
Overview of the collected samples and workflow. (**a**) Collection of five apples for analysis. (**b**) Experimental design and analytical pipeline for the study. Samples were collected from each apple at three stages of fruit development. Phenotypes were measured and RNA isolation was performed. Raw reads obtained from high-throughput to quality control and then mapped to the reference genome. Gene expression levels were quantified, and differential expression analysis was performed.

In order to investigate the correlation between gene expression and phenotypic variations in apples, we selected five distinct apple varieties that exhibit distinguishable phenotypes (Fig. 1a). Subsequently, we conducted measurements of 12 apple quality-related phenotypes, performed Illumina RNA-seq, and carried out bioinformatics analysis of the mRNA profile (Fig. 1b). Our study provides a comprehensive dataset comprising continuous phenomics data over a span of two years. Additionally, we obtained RNA-seq data for each apple at three different developmental stages, with three biological replicates. Bioinformatic analysis resulting in a total of 30,330 differential expression genes in pairwise comparisons among the samples at each stage. This dataset will serve as a valuable resource for researchers interested in investigating gene function and the mechanisms governing phenotypic variations in apples.

## Methods

### Sample collection

Five apple accessions were collected from the Institute of Pomology, Chinese Academy of Agricultural Sciences, located in Xingcheng, Liaoning Province. These accessions included three cultivated varieties (Weiqinni, Calville Rouge and Riskin) and two wild varieties (*M. sikkimensis* and *M. coronaria*). Samples were collected at various stages of fruit development, including fruitlet (S1), expanding (S2) and ripening (S3), which corresponded to 15–20, 60–70, and 105–135 days after flowering. Five fruits are selected from the four directions in the middle and the top center of the tree. Fruits were considered mature when they no longer showed an increase in size, exhibited fruit blush along with the disappearance of background color, and had a change in seed color from pale green to brown; these observations were combined with existing records of fruit maturity dates.

### RNA isolation and sequencing

Samples used for RNA sequencing were collected in 2014. Total RNA isolation was performed using the RNAprep Pure Plant Kit (TianGen, Beijing, China), following the guidelines provided by the manufacturer. Subsequently, it was adjusted to a concentration of 500 ng μL^−1^ using a NanoDrop Lite Spectrophotometer (Nanodrop Technologies, Wilmington, DE). The DNAase I (Takara, Dalian, China) was employed to eliminate any potential genomic DNA contamination during the RNA extractions. For library construction, 2 μg of RNA was utilized using the Illumina TruSeq RNA Kit in accordance with the manufacturer’s instructions. In total, 45 cDNA libraries from three development stages of five samples with three biological replicates were constructed for transcriptome sequencing. High-throughput RNA sequencing was performed by Illumina HiSeq 3000 platform (Illumina, San Diego, CA, USA), which obtaining paired-end sequencing data with a length of 150-bp.

### Measurement of phenotypes

Phenotypic measurements were continuously acquired during the years 2014 and 2015 on ripening fruits. Initially, the fruits were assessed for size and weight, followed by manual peeling, coring, and cutting into small pieces. Subsequently, they were promptly frozen using liquid nitrogen and stored at −40 °C for further analysis.

The transverse diameter (TD) and vertical diameter (VD) of the fruits were measured utilizing a vernier caliper. The weight of each fruit (FW) was determined by individually weighing them on a Mettler Toledo balance. The concentrations of organic acids (malate, citrate, oxalate, tartrate, and ascorbate) and soluble sugars (fructose, sucrose, glucose, sorbitol) were determined by high-performance liquid chromatography (HPLC) using an Agilent 1260 Infinity HPLC system (Milford, MA, USA) following previous reported method^17^.

### Data processing

The raw sequencing reads were filtered by removing adaptor sequences and trimming low-quality reads using fastp (version 0.21.0)^18^. Only reads with length more than 60 bp and no ambiguous (N) bases were kept for subsequent analysis. Clean reads were then aligned to the reference genome GDDH13(version 1.1)^19^ using HISAT2 (version 2.2.0)^20^ with default parameters. The output of HISAT2 was converted to binary format and sorted by samtools (version 1.12)^21^. The gene expression level was normalized as per kilobase million (TPM) and fragments per kilobase of exon per million fragments mapped (FPKM) by StringTie (version 2.1.4)^22^. Differentially expressed genes (DEGs) analysis were conducted using the DESeq 2 package^23^. The analysis utilized a read counts matrix output from the python script named prepDE.py, which is included with the StringTie software^22^.

## Data Records

The 45 raw RNA sequencing data (FASTQ) reported in this paper have been deposited in the NCBI database at Sequence Read Archive (SRA) under the project number PRJNA1037167^24^. Detailed SRA number of each file are listed in Table 1.

**Table 1:** Detailed SRA number of each sequencing file.

Varieties	Stages	Biological replicates		
		1	2	3
Weiqinni	Fruitlet (S1)	SRR26729862	SRR26729832	SRR26729870
	Expanding (S2)	SRR26729865	SRR26729859	SRR26729854
	Ripening (S3)	SRR26729848	SRR26729843	SRR26729837
Calville Rouge	Fruitlet (S1)	SRR26729851	SRR26729831	SRR26729869
	Expanding (S2)	SRR26729864	SRR26729858	SRR26729853
	Ripening (S3)	SRR26729847	SRR26729842	SRR26729836
Riskin	Fruitlet (S1)	SRR26729840	SRR26729830	SRR26729868
	Expanding (S2)	SRR26729863	SRR26729857	SRR26729852
	Ripening (S3)	SRR26729846	SRR26729841	SRR26729835
*M. sikkimensis*	Fruitlet (S1)	SRR26729874	SRR26729834	SRR26729872
	Expanding (S2)	SRR26729867	SRR26729861	SRR26729856
	Ripening (S3)	SRR26729850	SRR26729845	SRR26729839
*M. coronaria*	Fruitlet (S1)	SRR26729873	SRR26729833	SRR26729871
	Expanding (S2)	SRR26729866	SRR26729860	SRR26729855
	Ripening (S3)	SRR26729849	SRR26729844	SRR26729838

The phenomics data, normalized gene expression level matrix (TPM, FPKM and Counts), and differential expression genes (DEGs) list were deposited in FigShare (10.6084/m9.figshare.24522931.v2)^25^.

## Technical Validation

**Fig. 2:**
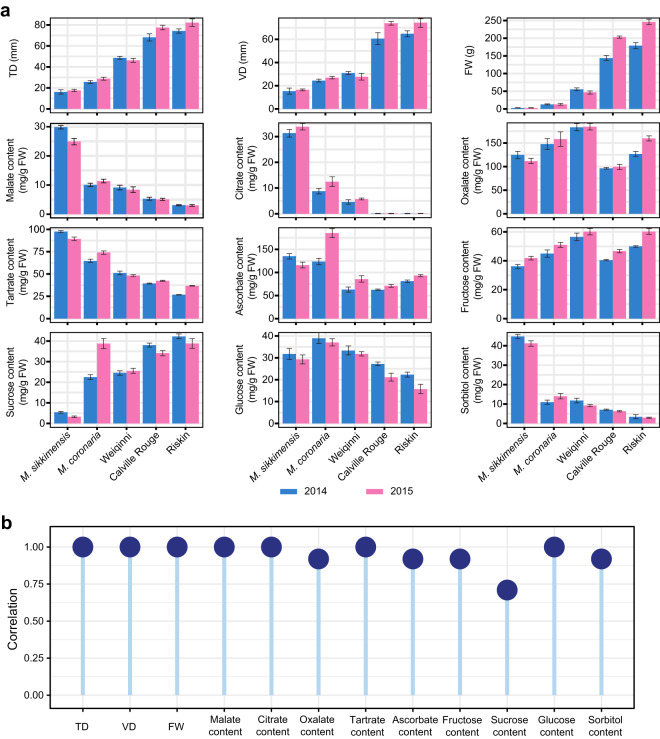
Phenomics data of five apples. (**a**) Twelve phenotypic values of five apples in 2014 and 2015. (**b**) Correlations of each phenotype between 2014 and 2015 (Spearman method, all P < 0.01).

The phenotypic dataset presented in this study comprises 12 distinct types, encompassing a range of critical measurements for characterizing fruit size, including transverse diameter (TD), vertical diameter (VD), and fruit weight (FW). Furthermore, the dataset incorporates several measurements of organic acid and soluble sugar contents, which play a significant role in determining the taste profile of fruits. These phenotypic traits exhibit discernible variations among different apple varieties, highlighting the diverse array of characteristics present within this species (Fig. 2a). To ensure precise measurement accuracy, a series of continuous measurements were conducted throughout the years 2014 and 2015. Statistical analysis revealed a significant correlation among the two-year measurements of each phenotype, as illustrated in Fig. 2b (Spearman’s rank correlation coefficient, P < 0.01). This correlation not only emphasizes the stable consistency observed within the phenomics dataset, but also serves to underscore its reliability as an essential resource for the comprehensive study of apple trait variations.

**Fig. 3:**
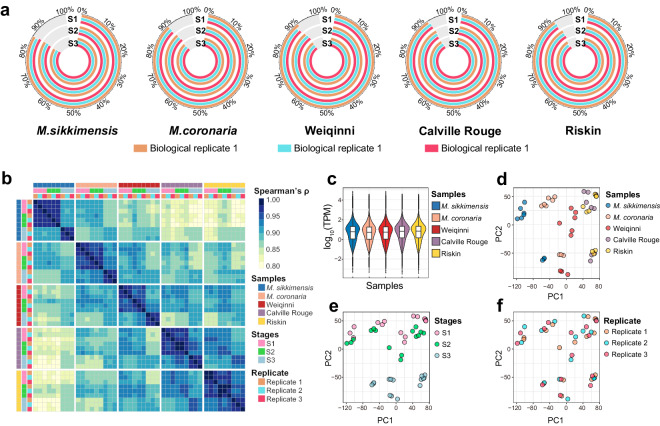
Transcriptomic data of five apples. (**a**) Mapping ratio of each sequencing library to the reference genome. (**b**) Correlation plot of all RNA-seq libraries. The columns and rows are the same, which are split by sample names, stages and replicates. Correlations were calculated by Spearman’s rank method. (**c**) Normalized expression levels of each gene in five apples. (**d–f**) PCA plots of all RNA-seq libraries for (**d**) samples (**e**) developmental stages and (**f**) biological replicate.

The transcriptomic dataset was generated in this study through the utilization of high-throughput sequencing on 45 cDNA libraries, resulting in the acquisition of approximately 298.01 gigabase (Gb) pair-end raw data. To ensure the accuracy and reliability of the data, a series of quality control steps were implemented, including adaptor sequences removal and elimination of low-quality regions at the beginning of each read as well as reads containing undetermined bases. Consequently, a set of approximately 228.29 Gb of clean data that passed the quality control measures was retained for further analysis. To validate the quality of clean reads, a meticulous assessment was performed using FastQC^26^, which unveiled that 96.92% of the clean bases exhibited a quality score surpassing the threshold of 30, indicating a remarkably low base error rate of less than 0.1%. This high-quality score suggests the accuracy of the obtained sequencing data. Furthermore, to evaluate the integrity and reliability of the sequencing data, the clean pair-end sequencing data were mapped to the apple reference genome titled ‘GDDH13 v1.1’^19^ using HISAT2^20^. The mapping results, represented in Fig. 3a, elucidated that 91.12% of the reads from each clean pair-end sequencing data were successfully mapped to the reference genome on average. This impressive mapping rate also provides additional evidence that enhances the reliability and accuracy of the obtained sequencing data. Meticulous preprocessing and rigorous quality assessment have yielded a substantial amount of clean and reliable data, which establishes a solid foundation for further investigate.

The reference genome version (GDDH13 v1.1) utilized for sequence mapping is a widely recognized resource in apple genome research^16,19,27–30^, encompassing transcript information for 45,116 protein-coding genes. To normalize the transcript quantities of each coding gene, the mapping results was normalized using TPM (Transcripts Per Million) and FPKM (Fragments Per Kilobase of transcript per Million mapped reads). Subsequently, correlation testing was conducted to assess the similarity between biological replicates from the same sample at a specific stage of development. The results demonstrated a strong similarity among these replicates, indicating a high level of reproducibility (Fig. 3b). The overall distribution of transcript expression across the three developmental stages showed similarity in each sample (Fig. 3c). Furthermore, a principal component analysis (PCA) was performed for all genes, enabling the visualization of distinct groupings of sequencing libraries from different samples and developmental stages. As shown in Fig. 1d,e, the presence of separate clusters suggests that there exists significant variability between samples and developmental stages. Conversely, it is noteworthy that biological replicates displayed a tendency to cluster together, indicating a high degree of repeatability (Fig. 3f). The utilization of the widely used reference genome version enabled researchers to normalize and analyze transcript quantities, thereby demonstrating comparability of gene expression levels in this dataset with others. Principal component analysis confirmed the presence of variability between samples and stages, while also highlighting the consistency and reproducibility of biological replicates.

**Fig. 4:**
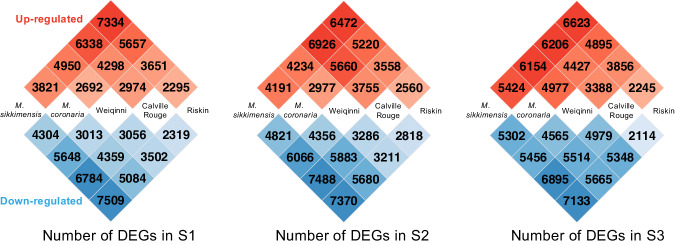
Differential expression genes analysis across five apples in each developmental stage. The numbers on the heatmap represent the count of differential expressed genes. Red represents up-regulated genes, while blue represents down-regulated genes.

Differentially expressed genes (DEGs) were identified using DESeq 2^23^ and genes meeting the defined criteria of having an absolute value of log2 fold change (FC) greater than 1.0 and an adjusted P-value lower than 0.05 were considered as DEG. To compare gene expression between different samples at the same developmental stage, paired analyses were conducted on the datasets. As depicted in Fig. 4, this analysis allowed for the identification of DEGs among different samples and revealing of distinct patterns. In the S1 and S3, *M. sikkimensis* and Riskin exhibited the highest number of DEGs. Whereas during the S2, *M. sikkimensis* and Calville Rouge were found to have the highest number of DEGs. By employing DESeq 2 for differential gene expression analysis, the DEGs across different samples and developmental stages were able to identify and characterized. These results help in understanding the variations in gene expression profiles and provides insights into the specific genes and sample combinations that exhibit notable differential expression patterns.

## Usage Notes

The dataset we released comprising of two types: phenomics and transcriptomics. The phenomics data encompasses two files in csv format, each containing the phenotypic data recorded for the years 2014 and 2015, respectively. The row names in these files correspond to various traits, while the column names represent the individual samples, facilitating convenient analysis. Fruit size related traits (TD and VD) are in millimeters (mm). Fruit weight (FW) is in grams (g). The organic acids and soluble sugars are in milligrams per gram (mg/g).

For the transcriptomic data, we have included both the raw dataset available in fastq format, as well as the processed data presented in csv format. The processed data consists of normalized expression values, specifically the TPM and FPKM. Additionally, we have incorporated a list of DEGs for each pair of samples. Moreover, to further assist users in performing custom DEG analysis, we have included a normalized read counts matrix that can be readily imported into the DESeq 2 package^23^, which store the phenomics data of 2014 and 2015 respectively. The row names (traits) and column names (samples names) are friendly marked which are convenient for users to read for analyzing. The transcriptomic data includes raw dataset in fastq format and processed data in csv format. The processed data including normalized expression values (TPM and FPKM) and DEGs list of each sample pairs. Additionally, we also provided normalized read counts matrix which users can easily import to DESeq 2^23^ for custom DEG analysis.

## Data Availability

The following are the commands for data processing. The analysis is deployed on CentOS 7 platform. All software versions have been specified in the Methods section. The reference genome version we used is GDDH13_v1.1, detailed annotation and gene prediction information can be found here (https://www.rosaceae.org/species/malus/malus_x_domestica/genome_GDDH13_v1.1). 1. Quality control $ fastp -i sample_raw_1.fq.gz -o sample_clean_1.fq.gz -I sample_raw_2.fq.gz -O sample_clean_2.fq.gz -r --length_required 60 -f 12 2. Read mapping $ hisat2 --dta --summary-file sample.summary.txt --new-summary --min-introlen 20 --max-introlen 5000 reference.genome -1 sample_clean_1.fq.gz -2 sample_clean_2.fq.gz -S sample.sam 3. Convert and sort $ samtools sort sample.bam sample.sam 4. Normalize $ stringtie -G reference.gff3 -e -B -o sample.gtf -A sample.tab sample.bam
